# Severity and Treatment Difficulty of Impacted Maxillary Canine among Orthodontic Patients in Riyadh, Saudi Arabia

**DOI:** 10.3390/ijerph191710680

**Published:** 2022-08-27

**Authors:** Laila Fawzi Baidas, Nada Alshihah, Rwan Alabdulaly, Sara Mutaieb

**Affiliations:** 1 Department of Pediatric Dentistry and Orthodontics, College of Dentistry, King Saud University, Riyadh 11451, Saudi Arabia; 2 College of Dentistry, King Saud University, Riyadh 11451, Saudi Arabia

**Keywords:** impaction, maxillary canines, severity, difficulty index

## Abstract

Background: The current study aimed to evaluate the severity and treatment difficulty of impacted maxillary canines and their relationship with gender, age group, and bucco-palatal position. Methods: A retrospective cross-sectional study was conducted from 2017 to 2021. Patients’ data and panoramic radiography were obtained from the orthodontic clinic at King Saud University’s Dental University Hospital in Riyadh, Saudi Arabia. The severity factors and treatment difficulty index were used to assess the impacted maxillary canines. Statistical analyses were performed utilizing a chi-square test for categorical variables and an independent *t*-test for numerical variables, and a *p*-value of ≤0.05. Results: There were 171 impacted maxillary canines in total, with a female-to-male subject ratio (11:8) and a mean age (18.7 years). Overall, 77.2% of impacted upper canines were found to be palatal. The severity of canine impaction parameters showed no significant sex or age group predilection. Buccally impacted maxillary canines were characterized by a preferable angulation to the midline, compared to the palatally impacted maxillary canines (*p* = 0.012). The horizontal overlap of the impacted maxillary canine cusp tip revealed a significant association with the bucco-palatal position of the impacted canine (*p* < 0.001). Palatal impaction was located more frequently in sectors 3 and 4. Male patients were found to have a higher total score in terms of the treatment difficulty index relative to females (*p* = 0.046). Conclusion: Despite the severity parameters having revealed no significant gender predilection, males were found to have higher treatment difficulty in maxillary canine impaction than females. The severity of the palatally impacted canine is greater than that of buccal impaction in terms of angulation to the midline and horizontal overlap.

## 1. Introduction

Maxillary canine impaction (IMC) is the most common type of impaction, following that of the third molars [1,2]. The prevalence of maxillary canine impaction is stated to range between 1% and 3% across varying populations [3,4,5,6]. While the etiology of IMC remains unclear and is multifactorial, genetic factors certainly contribute to the development of palatally displaced canines, and local factors are more frequently associated with buccally displaced canines. The latter factors include a lack of space to accommodate the erupting canines, malformed lateral incisors, or missing lateral incisors, which result in a lack of guidance to the erupting canines [7,8,9]. Early diagnosis of unerupted maxillary canines remains essential to impaction detection. If the labial canine bulge above the maxillary canines is missing in patients older than ten years old, impaction may be expected [10]. 

A variety of radiographic techniques are used to evaluate the presence, position, and pathology related to impacted canines, such as intra-oral periapical radiograph, occlusal radiograph, panoramic radiograph, computed tomography (CT), and, lastly, cone-beam computed radiography (CBCT) [9,10]. CBCT is a three-dimensional imaging modality that offers a reliable method to accurately locate the impacted canine. However, it may expose the patient to more radiation, may not be accessible in every treatment center, and cannot be used for early assessment [11,12]. Panoramic radiography (OPG) is considered to be one of the most reliable imaging tools as it allows a thorough two-dimensional view of the impacted teeth in terms of their position, angulation, and orientation relative to adjacent teeth [13].

The precise localization of the IMC can aid in the orthodontic treatment decision to either expose and orthodontically align or remove the IMC. Several classification systems are available for assessing the severity of maxillary canine impaction on a single panoramic radiograph. The sector technique was developed by Ericson and Kurol and modified by Lindauer et al. to determine the horizontal position of the canine tip in relation to the root of the lateral incisor [14,15]. The vertical position of the impacted canine crown from the occlusal plane and the angulation of the impacted canine axis to the midline was described by Short and Power [16]. Ackerman and Fields described impacted canines as horizontal, relative to the arch, and vertical, relative to the apex [17]. As the orthodontic alignment of an IMC to its normal position may require lengthy and complex treatments, several prognostic indices for estimating the severity of the treatment of impacted canines have been reported in the literature. However, currently, success predictions are based heavily on clinical experience and anecdotal evidence [18,19,20]. Pitt et al. developed a treatment difficulty index based on nine parameters that influence the treatment difficulty in descending order of priority, thereby offering a useful treatment planning tool for the management of impacted canines [19]. A study conducted in Jizan, Saudi Arabia, used the Pitt et al. index to assess the treatment difficulties of IMC, identifying the treatment difficulty index to be higher in males than females [20].

As practitioners, we are interested in examining the pattern of impacted canines and determining the potential factors that affect the severity of impaction and, in turn, the difficulty of the treatment required. This study aimed to evaluate the severity and the treatment difficulty index of maxillary canine impaction, compare the severity of impacted canines with regard to gender, age groups, and bucco-palatal position, and compare treatment difficulty index values with regard to gender.

## 2. Materials and Methods

A retrospective cross-sectional study was conducted involving OPG images for orthodontic patients at the Dental University Hospital in Riyadh, Saudi Arabia, during 2017–2021. OPG images were routinely obtained for pretreatment diagnostic records but not for the study purpose. Ethical approval (IRB No. E-21-6411) was obtained from the review board at the College of Medicine, King Saud University, Saudi Arabia. A single examiner screened all OPG images for the presence of impacted canine(s). The screened images were then filtered using the inclusion and exclusion criteria. The inclusion criteria were as follows: patients between 14 and 30 years of age, the presence of impacted permanent maxillary canines and lateral incisors, and the presence of a diagnostic cast. The exclusion criteria were as follows: the presence of any pathological condition in the area of interest that could affect the measurement, syndrome, or cleft lip and palate patients. Patients’ data regarding age, gender, and the buccolingual position of the impacted canine were obtained from the SALUD system.

### 2.1. Panoramic Radiographs

The sample size was calculated using chi-square at alpha 0.05 and power 0.95. Hence, at least 124 radiographs were required. Digital panoramic images were taken using ORTHOPHOS XG 3 (Sirona Dental Systems GmbH, Wals bei Salzburg, Austria) with a magnification factor of 1:1. All measurements were adjusted according to this magnification factor. Panoramic radiographs were digitized (JPEG format) using an Epson Perfection V700 Photo Scanner (Epson America, Inc., Long Beach, CA, USA). Romex software (PLANMECA USA, Illinois, and USA) was used to determine lines in each panoramic image. Ten panoramic radiographs were assessed by two validated examiners at two-week intervals to evaluate intra- and inter-examiner measurement reliability.

The severity score of maxillary canine impaction was evaluated according to the classification system proposed in the literature [14,15,20].

**Figure 1:**
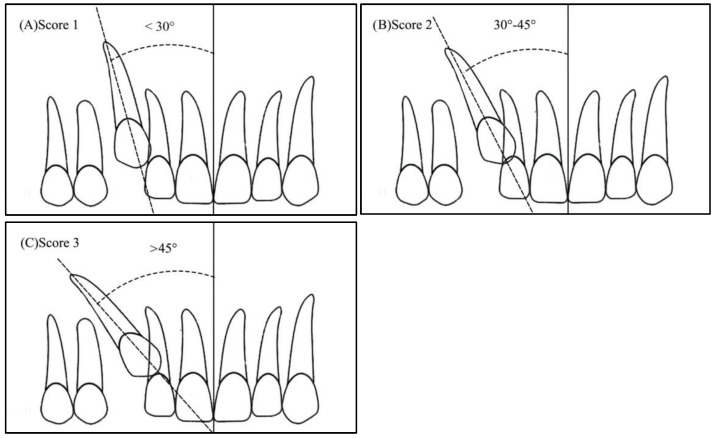
Angulation of the impacted canine to the midsagittal plane: (**A**) Score 1—less than 30 degrees, (**B**) Score 2—between 30 and 45 degrees, (**C**) Score 3—more than 45 degrees.

**Figure 2:**
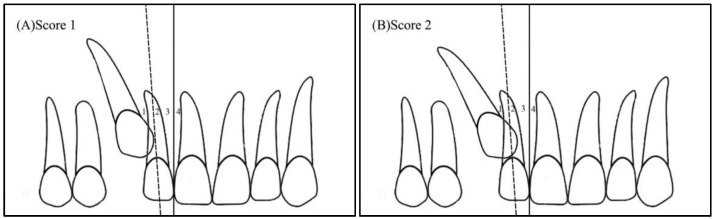

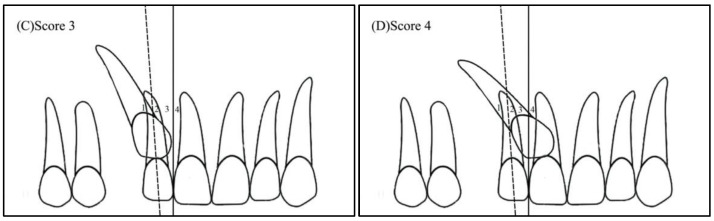
Horizontal position of the impacted canine: (**A**) Sector 1—canine overlapping less than half the width of the lateral incisor, (**B**) Sector 2—canine overlapping half the width of the lateral incisor, (**C**) Sector 3—canine completely overlapping the lateral incisor, (**D**) Sector 4—enclosing all areas mesial to Sector 3.

**Figure 3:**
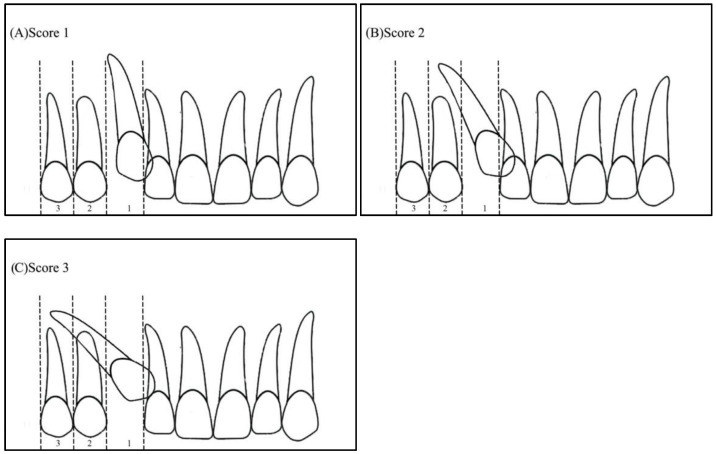
Apex position: (**A**) score 1—in the area of canine apex, (**B**) score 2—in the area of the first premolar apex, (**C**) score 3—in the area of the second premolar apex.

**Figure 4:**
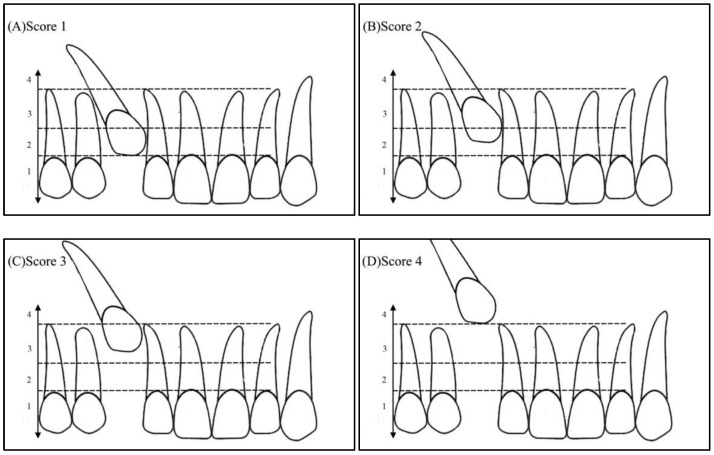
Vertical position of the impacted canine: (**A**) Score 1—canine cusp tip at the level of the cementoenamel junction of the adjacent incisor, (**B**) Score 2—canine cusp tip at the middle of the root of the adjacent incisor, (**C**) Score 3—canine cusp tip within the apical third of the root of the adjacent incisor, (**D**) Score 4—canine cusp tip above the apical third of the root of the adjacent incisor.

1. Angulation between the midsagittal plane and the long axis of the impacted tooth is as follows: score 1—mild (<30°), score 2—moderate (30°–45°), and score 3—severe (˃45°) (Figure 1);
2. Horizontal overlap: Sector 1 (canine overlapping less than half the width of the lateral incisor), Sector 2 (canine overlapping half the width of the lateral incisor), Sector 3 (canine completely overlapping the lateral incisor), and Sector 4 (enclosed all areas mesial to Sector 3) (Figure 2);
3. Apex position: score 1 (in the area of canine apex), score 2 (in the area of the first premolar apex), and score 3 (in the area of the second premolar apex) (Figure 3);
4. Vertical overlap: score 1 (canine cusp tip at the level of the cementoenamel junction of the adjacent incisor), score 2 (canine cusp tip at the middle of the root of the adjacent incisor), score 3 (canine cusp tip within the apical third of the root of the adjacent incisor), and score 4 (canine cusp tip above the apical third of the root of the adjacent incisor) (Figure 4).

### 2.2. Treatment Difficulty Index for Unerupted Maxillary Canine

The Pitt et al. index was used to assess the treatment difficulty index for unerupted maxillary canines [19]. The following factors were assessed: patient age, angulation to the midline, vertical position, buccolingual position, horizontal position, incisor alignment, canine space, midline coincidence, and the rotation of the impacted tooth. The grading system and weighting factors of each variable are presented in Table 1. The weighting of each factor in the difficulty score was set according to that proposed by Pitt et al. [19]. All factors corresponded to a set of variables with designated scores, while each factor had a specific weight. Each factor score was multiplied by the validated weight. The total score ranged between 10.5 and 33.5, with the higher values identifying higher treatment difficulty [20].

### 2.3. Statistical Analysis

Statistical analysis was conducted using SPSS 26.0 version (IBM Inc., Chicago, IL USA), a statistical software package. The level of significance was set at a *p*-value ≤ 0.05.

1. Descriptive statistics (mean, standard deviation, frequencies, and percentages) were used to quantify the quantitative and categorical variables;
2. Pearson’s chi-square test, followed by the deviance test, was used to observe the associations between the categorical variables;
3. An independent *t*-test was used to examine gender differences in the treatment difficulty index.

## 3. Results

A total of 3350 OPG images were screened. Of the population that corresponded with these images, 171 individuals with maxillary impacted canines were identified as satisfying the inclusion criteria, with 99 female (57.9%) and 72 male (42.1%) patients. The patients’ mean age was 18.7 ± 4 years old, and the participants were further divided into the following two age groups: the younger age group (14–18 years, 60.2%) and the older age group (19–30 years, 39.8%). The majority of impacted upper canines (77.2%) were palatal, with only 22.8% being buccal. Furthermore, a slight majority of the impacted canines (52%) were on the left side, as opposed to the right side (48.9%). Females were found to represent a higher proportion within the 14–18 age group as compared with the male subjects of the same age group (*p* = 0.044). However, no significant difference was found between males and females in the distribution of impacted upper right or left canines, or buccal and palatal canine impaction on the right and left sides (See Table 2).

The repeated measurements of ten selected cases revealed good reliability, with inter-class correlation coefficients ranging between 0.835 and 0.976 for intra-examiner reliability and 0.838 and 0.973 for inter-examiner reliability.

### 3.1. The Severity of the Impacted Canine

The descriptive statistics of the severity score of maxillary canine impaction, as well as the comparisons across gender, age groups, and bucco-palatal positions, are given in Table 3. Only 34% of impacted canines were significantly angulated to the midline (>45°), whereas 39% were angulated <30° to the midline. The majority of impacted canines (41.5%) overlapped less than half of the lateral incisor root (Sector 1), while 26.9% overlapped up to half of the central incisor root (Sector 4). Fifty-nine percent of the impacted canines had their root apexes at the first premolar apex position. The cusp tip of impacted canines was located in the middle of the adjacent incisors in 65.5% of the totally impacted canines. 

**Figure 5:**
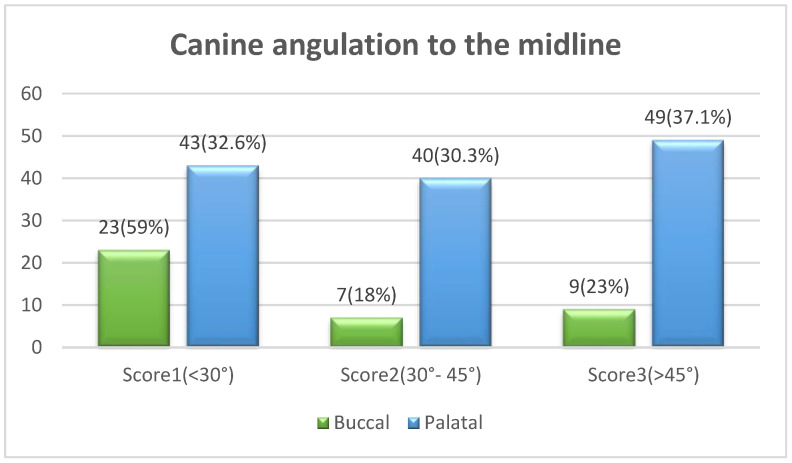
Comparison between buccal–palatal canine impaction and the canine angulation to the midline using the chi-square test (X^2^ = 40.7, df = 3, *p* < 0.001 *). * Statistically significant at *p* ≤ 0.05.

**Figure 6:**
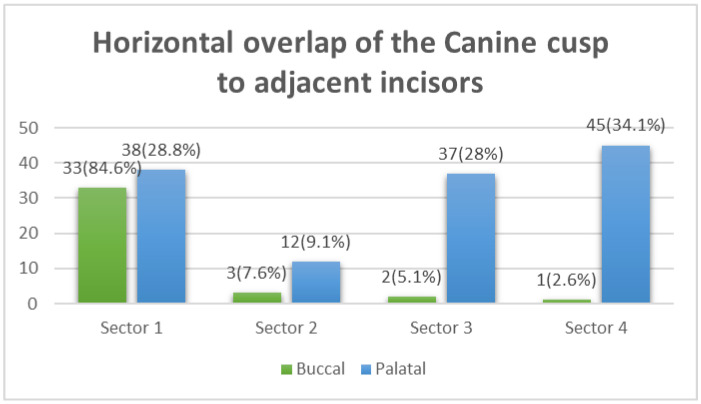
Comparison between buccal–palatal canine impaction and the horizontal overlap of the impacted canine crown tip with adjacent teeth using the chi-square test (X^2^ = 40.7, df = 3, *p* < 0.001 *). * Statistically significant at *p* ≤ 0.05.

The chi-square test revealed no statistically significant differences in the distribution of maxillary canine impaction severity scores across genders or age groups. However, canine angulation to the midline showed a statistically significant difference (*p* = 0.012) in terms of the bucco-palatal canine position. Fifty-nine percent of buccal IMC cases were characterized by mild angulation to the midline (<30 degrees), while the palatal IMC showed a higher percentage (67.4%) of moderate-to-severe angulation to the midline (Figure 5). Similarly, the horizontal overlap of the IMC cusp tip (sectors) revealed a significant difference with respect to the bucco-palatal position of the impacted canine (*p* < 0.001). Buccally impacted canines were more likely to be found in Sector 1, whereas palatal impaction was more likely to be observed in Sectors 3 and 4 (Figure 6).

### 3.2. The Total Treatment Difficulty Index

The descriptive values of the weighted scores of the treatment difficulty index, in addition to the differences across genders, are shown in Table 4. The mean score of the age of the subjects (4.68 ± 1.2), vertical position (2.91 ± 0.94), horizontal position (4.7 ± 0.85), and canine space available (2.73 ± 0.85) were all found to be significantly high overall. No statistically significant differences were found across male and female subjects for each component of the treatment difficulty index. However, male subjects showed a higher total treatment difficulty score compared to female subjects (*p* = 0.046).

## 4. Discussion

A thorough understanding of the severity of maxillary canine impaction and the anticipated treatment difficulty is expected to significantly benefit the diagnosis, selection of management strategies, and estimation of treatment costs and time. A few studies in Saudi Arabia have evaluated the severity of impaction and the difficulty of orthodontic therapy [1,2,19]. This study aimed to evaluate the severity of the impacted canine and the treatment difficulty index of maxillary canine impaction and their relation to gender, age group, and bucco-palatal position.

Maxillary canine impaction in females was found to be higher than in males, which aligns with other national and global studies [2,20,21,22]. This finding may be attributed to the variance in craniofacial growth and developmental influences between males and females, or the higher esthetic demand of females compared to males [21]. The present study found maxillary canine impaction to be significantly higher in the younger female group (*p* = 0.004). Moreover, palatal impaction (77.2%) was found to be more prevalent than buccal impaction (22.8%), as reported in the majority of other studies [20,21,22,23]. In contrast with previous studies that found a higher prevalence of impacted maxillary canines on the left side than on the right side [24,25], our findings revealed a nearly similar distribution of impacted maxillary canines on both sides (52% on the right and 48.9% on the left). At present, there remains no empirical evidence to explain the difference in prevalence across the right and left sides. Thus, it may be considered a general feature of malformation.

Many studies have developed a grading system to determine the severity of canine impaction [14,15,20]. The following four main radiographic parameters are included in this grading system: the angulation of the canine long axis to the midline, the vertical position of the canine crown from the occlusal plane, the anteroposterior position of the canine root apex, and the degree of overlap of the adjacent incisor by the canine crown tip. 

The angulation of the maxillary canine to the midsagittal plane is directly related to the severity of impaction. In the present study, females were found to have a larger number of impactions with mild angulation (<30°) to the midline, while males had a larger number of impactions with severe angulation (>45°); however, there was no statistical difference between males and females, which is consistent with the study of Alhammadi et al. [20], where no gendered difference was found in terms of the impacted tooth angulation. In contrast, Alabdullah et al. found that females had a larger impaction angle than males and that females were generally characterized by more severe impaction of the teeth, and the maxillary canine in particular [14]. When comparing age groups in terms of the angulation of impacted canines, the angulation of the canine increased with age; however, this difference was not statistically significant. It was revealed that the angle of impaction also increased with age, highlighting the significance of early detection and intervention [14]. With regard to the IMC bucco-palatal position and its relationship with the angulation to the midline, palatal impaction was found to have significantly higher angles to the midline (*p* = 0.012) than buccal impaction. This finding is similar to that of another study, which found palatal IMC to have a greater root angle towards the midsagittal plane [24]. 

In terms of the horizontal position of the maxillary canine impaction, sector localization is used to locate the tip of the impacted canine in the mesial–distal direction by drawing lines that differ for varying systems of sector locators introduced by researchers. Lindauer et al. modified the Ericson and Kurol classification of five sectors and reduced it to four [14,15]. We implemented the four-sector approach in this study due to its convenience, simplicity, and rapid production of results. Our findings showed that 71.5% of the total sample of IMC cases were located in Sector 1; 84.6% were labially located, while 28.8% were palatally located, which contradicts the findings of Lindauer et al. [15]. However, the finding also remains inconsistent with that of Alfaleh and Al Thobiani, who stated that none of the palatally impacted canines were found in Sector 1 [25]. Furthermore, their study revealed that impacted canines in Sector 2 primarily tend to be located in the labial area, which is contrary to our findings, which revealed the majority of them to be located palatally. The majority of impacted canines in Sector 3 (22.8%) and Sector 4 (26.9%) were located palatally, which is consistent with the findings of Alfaleh and Al Thobiani [25]. This variation in the study findings may be attributed to the use of different methods to determine the impaction as well as the varying radiograph techniques employed. The comparison between males and females, and between older and younger age groups, in terms of the horizontal position of the impacted canine was not statistically significant, which is in agreement with Alhammadi et al. [20].

The apex position of the impacted maxillary canine is a predictor of impaction severity. In our study, the IMC apex was mainly found in the first premolar area, which is in line with the extant literature [20,21,22,23,24,25,26]. However, the apex position demonstrated relatively close percentages, with no statistically significant difference across gender, age group, and buccolingual position.

Regarding the vertical position of the IMC relative to the root of the adjacent incisor, it was found that the IMC was most frequently positioned between the cementoenamel junction and root apex [27]. Another study showed that 83.78% of labial and 50% of palatal impacted canines are positioned in the coronal zone of the root [28]. Similarly, our results found that most of the IMC cases were located in the area between the cementoenamel junction up to the middle of the root. However, the vertical position of the IMC across gender, age, and bucco-palatal position groups showed no significant difference, which is in line with the study of Alhammadi et al.; however, these findings contradict Alabdullah et al., who found that the vertical position of the canine worsens with age but does not reach the level of statistical significance [18,19,20]. The relation between the vertical position of the canine, treatment difficulty, and duration is well known. Fleming et al. found that the treatment duration increased by 6 months if the cusp tip of the impacted canine was positioned more than halfway above the adjacent tooth [13]. A recent paper by Gunardi et al. found that the treatment decision is affected by the vertical position and that most of the teeth require surgical removal instead of the forced eruption when the cusp tips are located at the apical root of the incisor [29].

The complexity and treatment difficulty of IMC are influenced by several clinical and radiographic factors [30,31]. Pitt et al. curated a variety of these factors and developed a single index to measure treatment difficulty; they stated that the horizontal position, patient age, vertical height, and bucco-palatal position (in descending order of importance) are factors that determine the difficulty of impacted canine treatment [19]. Upon comparing TDI scores between males and females, individual factors of the index did not show a statistical difference. However, a significant difference was found in the total score of the TDI, with males having higher scores and, consequently, requiring more difficult treatment. This was in agreement with Alhammadi’s study, where the same TDI was used to determine differences in treatment difficulty across gender groups [20]. Moreover, their study identified a statistically significant difference between males and females in terms of certain individual components of the TDI (angulation to the midline, vertical position, alignment of the upper incisors, and midline coincidence). 

The treatment severity grading and treatment difficulty index applied in this research are clinically relevant to identifying the degree of impaction and developing an appropriate treatment plan. A panoramic radiograph was utilized for this purpose due to its accessibility and ease of interpretation. However, the panoramic radiograph is a two-dimensional image of a three-dimensional structure, which can pose certain limitations. Crowding, tooth rotation, labiolingual connection, precise root angulation, and closeness to adjacent teeth are all variables that cannot be accurately determined. Hence, clinical evaluation is recommended to correlate the findings of the panoramic radiograph. Further studies are recommended to determine the severity and treatment index of impacted canines using cone-beam computed tomography, which may then be compared with the findings of the current study. 

## 5. Conclusions

The main findings of this study are summarized as follows:

1. Maxillary canine impaction was higher in younger females (14–18 y) compared to males of the same age;
2. The severity of IMC was not found to be different between males and females, or across age groups;
3. Palatal impactions were found to be more severe than buccal impactions due to the worsening horizontal position and larger angulation to the midline;
4. The horizontal overlap of the IMC cusp tip (sectors) revealed a significant association with the bucco-palatal position of the impacted canine. Most of the buccally positioned IMC cases were located in Sector 1, while over two thirds of the palatally positioned impactions were located in Sectors 3 and 4;
5. Although canine impaction was found to be higher in females, males had higher treatment difficulty index scores and, thus, were characterized by relatively higher expected treatment difficulty.

## Figures and Tables

**Table 1:** Grading score and weighting of the orthodontic treatment difficulty index.

Factors	Score	Weight
**Age**		
Less than 12 years	1	1.5
12–15 years	2	
15–18 years	3	
Over 18 years	4	
**Angulation to midline**		
Less than 30 degrees	1	1
30–45 degrees	2	
Over 45 degrees	3	
**Vertical position**		
Canine cusp tip at the level of the amelocemental junction of the adjacent incisor	1	1.5
Canine cusp tip at the middle of the root of the adjacent incisor	2	
Canine cusp tip within the apical third of the adjacent incisor	3	
Canine cusp tip above the apical third of the adjacent incisor	4	
**Bucco-palatal position**		
Buccal	1	1
Palatal	1	
**Horizontal position**		
Canine overlapping less than half the width of the lateral incisor	1	2
Canine overlapping over half the width of the lateral incisor	2	
Canine completely overlapping the lateral incisor	3	
Canine overlapping up to half the width of the central incisor	4	
**Alignment of upper incisors**		
Incisors spaced	1	0.5
Incisors well-aligned	2	
Incisors crowded	3	
**Space between the upper lateral incisor and upper first premolar**		
Over 7 mm	1	1
4–7 mm	2	
2–4 mm	3	
0–2 mm	4	
**Midline**		
Midline coincident with lower	1	1
Midline displaced	2	
**Rotation**		
Rotation absent	1	1
Rotation present	2	

**Table 2:** Distribution and comparison of age and impacted canines between male and female study subjects (N = 171).

Variable		Total		Male		Female		
		No.	%	No.	%	No.	%	*p*-Value
Age groups	14–18	103	60.2	37	51.4	66	66.7	0.044 *
	19–30	68	39.8	35	48.6	33	33.3	
Impacted upper canine side	Right	89	52	41	56.9	48	48.5	0.274
	left	82	48	31	43.1	51	51.5	
Bucco-lingual position	Buccal	39	22.8	17	23.6	22	22.2	0.486
	Palatal	132	77.2	55	76.4	77	77.8	

* Statistically significant at *p* ≤ 0.05.

**Table 3:** Associations between the severity of canine impaction, gender, age group, and bucco-palatal impaction using Pearson’s chi-square test of independence (N = 171).

Parameters	Score		Gender			Age Group (Years)			Bucco-Lingual Position		
		Total	Male	Female		14–18	19–30		Buccal	Palatal	*p*-Value
		No (%)	No (%)	No (%)	*p*-Value	No (%)	No (%)	*p*-Value	No (%)	No (%)	*p*-Value
Tooth angulation to the midline	<30°	66 (38.6)	24 (33.3)	42 (42.4)	0.185	43 (41.7)	23 (33.8)	0.402	23 (59)	43 (32.6)	0.012 *
	30–45°	47 (27.5)	18 (25.0)	29 (29.3)		29 (28.2)	18 (26.5)		7 (18)	40 (30.3)	
	>45°	58 (33.9)	30 (41.7)	28 (28.3)		31 (30.1)	27 (39.7)		9 (23)	49 (37.1)	
Horizontal overlap of the canine cusp to adjacent incisors	<1/2 of the lateral incisor	71 (41.5)	26 (36.1)	45 (45.5)	0.418	42 (40.8)	29 (42.6)	0.705	33 (84.6)	38 (28.8)	<0.001 **
	Up to 1/2 of the lateral incisor	15 (8.8)	6 (8.3)	9 (9.1)		11 (10.7)	4 (5.9)		3 (7.7)	12 (6.3)	
	>1/2 of the lateral incisor	39 (22.8)	16 (22.2)	23 (23.2)		24 (23.3)	15 (21.1)		2 (5.1)	37 (28)	
	Up to 1/2 of the central incisor	46 (26.9)	24 (33.3)	22 (22.2)		26 (25.2)	20 (29.4)		1 (2.6)	45 (34)	
Apex position	Canine position	8 (4.7)	3 (4.2)	5 (5.1)	0.643	5 (4.8)	3 (4.4)	0.907	0 (0)	8 (6.1)	0.098
	First premolar	101 (59.1)	40 (55.6)	61 (61.6)		62 (60.2)	39 (57.4)		28 (71.8)	73 (55.3)	
	Second premolar	62 (36.3)	29 (40.3)	33 (33.3)		36 (35.0)	26 (38.2)		11 (28.2)	51 (38.6)	
Vertical position relative to the adjacent incisor	At the level of CEJ	36 (21.1)	14 (19.4)	22 (22.2)	0.495	22 (21.4)	14 (20.6)	0.170	9 (23.1)	27 (20.5)	0.752
	In the middle of the root	112 (65.5)	50 (69.4)	62 (62.6)		70 (68.0)	42 (61.8)		23 (58.9)	89 (67.4)	
	Within the apical third	20 (11.7)	6 (8.3)	14 (14.1)		11 (10.7)	9 (13.2)		6 (15.4)	14 (10.6)	
	Above the apex	3 (1.8)	2 (2.8)	1 (1.0)		0	3 (4.4)		1 (2.6)	2 (1.5)	

* Statistically significant at *p* ≤ 0.05.

**Table 4:** Comparison of the components of the difficulty index between male and female study subjects using an independent *t*-test (N = 171).

Components of the Difficulty Index (Weighted Scores)	Total		Male		Female		
	Mean (±SD)	Range	Mean (±SD)	Range	Mean (±SD)	Range	*p*-Value
Age	4.68 (1.2)	1.5–6	4.81 (1.3)	1.5–6	4.59 (1.2)	3–6	0.246
Angulation to the midline	1.95 (0.85)	1–3	2.08 (0.9)	1–3	1.86 (0.8)	1–3	0.089
Vertical position	2.91 (0.94)	1.5–6	2.92 (0.9)	1.5–6	2.91 (1)	1.5–6	0.959
Bucco-lingual position	1	1	1	1–1	1	1–1	0
Horizontal position	4.7 (0.85)	2–8	5.06 (2.5)	2–8	4.44 (2.5)	2–8	0.12
Alignment of the upper incisors	0.88 (0.28)	0.5–1.5	0.9 (0.3)	0.5–1.5	0.86 (0.3)	0.5–1.5	0.368
Canine space	2.73 (0.85)	1–4	2.72 (0.9)	1–4	2.73 (0.8)	1–4	0.97
Midline	1.77 (0.42)	1–2	1.79 (0.5)	1–2	1.76 (0.4)	1–2	0.602
Rotation	1.51 (0.50)	1–2	1.57 (0.5)	1–2	1.47 (0.5)	1–2	0.178
Total difficulty index	22.13 (4)	14.5–31.5	22.85 (3.8)	15.5-31	21.62 (4)	14.5–31.5	0.046 *

* Statistically significant at *p* ≤ 0.05.

## Data Availability

3rd Party Data Restrictions apply to the availability of these data. Data was obtained from [Dental University Hospital in Riyadh, Saudi Arabia. OPG and cephalometric radiographs, and orthodontic study models. Ethical approval (IRB No. E-21-6411) was obtained from the review board at the College of Medicine, King Saud University, Saudi Arabia].
